# Electronically-augmented Huddle Improved Antibiotic Delivery for Sepsis in the Pediatric Intensive Care Unit

**DOI:** 10.1097/pq9.0000000000000059

**Published:** 2018-04-17

**Authors:** Charlotte Z. Woods-Hill, Lauren M. Biedron, Valerie A. Rigby, Sharon Kaminski, Kelly E. Papili, Julie C. Fitzgerald, Scott L. Weiss

**Affiliations:** From the * Division of Critical Care Medicine, Children’s Hospital of Philadelphia, Philadelphia, Pa.; ‡ Children’s Hospital of Philadelphia, Philadelphia, Pa.; † Leonard Davis Institute of Health Economics, University of Pennsylvania, Philadelphia, Pa.

## Abstract

**Background:**

Delayed antimicrobial therapy is an independent risk factor for mortality and prolonged organ dysfunction in sepsis. Barriers to timely antibiotic administration include lack of situational awareness about medication status and inconsistent communication with ordering clinicians.

**Objectives:**

The specific aim of this project was to achieve antibiotic administration within 60 minutes of order for ≥ 70% of suspected sepsis episodes in pediatric intensive care unit patients.

**Methods:**

In the 55-bed pediatric intensive care unit of an academic referral center, a standardized workflow to decrease antibiotic administration time for patients with suspected sepsis was iteratively implemented from 2012 to 2017. An electronic orderset (phase 1) and best practice alert for “stat” antibiotic ordering (phase 2) were combined with a scripted multidisciplinary bedside “sepsis huddle” (phase 3). Subsequently, a bedside, 1-touch electronic notification button was introduced (phase 4), which triggered automated phone alerts to the clinical team until antibiotic administration was complete (Fig. 1).

**Results:**

There was a progressive decrease in time from antibiotic order to administration from phase 1 through 4 (Table 1; Fig. 2). This improvement has been sustained, with ≥ 70% of suspected sepsis episodes meeting goal antibiotic administration time for 12 months following phase 4. On-time administration was more likely for episodes with versus without a huddle (90% versus 70%).

**Conclusions/Implications:**

Combining automated, timed reminders with a multidisciplinary huddle improved situational awareness about challenges to timely antibiotic delivery and decreased time to administration for critically ill children with suspected sepsis. Follow-up work includes integrating the 1-touch notification process into an automated sepsis recognition algorithm.

**Fig. 1.**
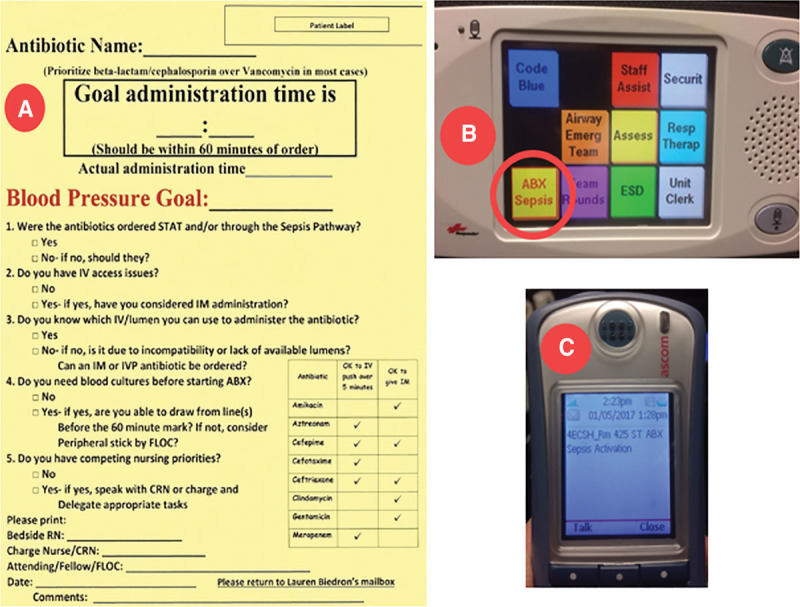
A, Sepsis huddle script. B, One-touch notification button. C, Alert to clinician.

**Fig. 2.**
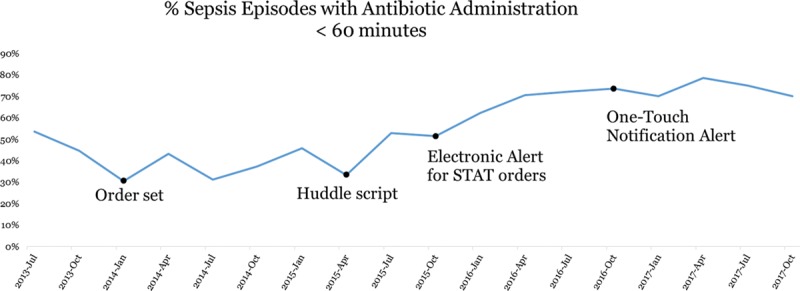
Percentage of suspected sepsis episodes meeting goal antibiotic administration time during phases 1–4.

**TABLE 1.**
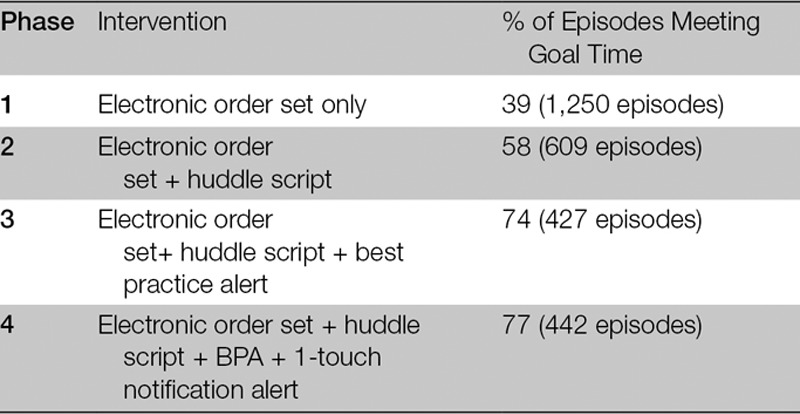
Improvement in Antibiotic Administration with Each Phase of Intervention

